# Changes in Antisecretory Factor in Human Milk During the Postpartum and Length of Gestation

**DOI:** 10.1177/08903344211021306

**Published:** 2021-06-01

**Authors:** Anna Gustafsson, Ewa Johansson, Ewa Henckel, Stefan Lange, Kajsa Bohlin

**Affiliations:** 1 27106 Department of Neonatology, Karolinska University Hospital, Stockholm, Sweden; 2 206106 Department of Clinical Science, Intervention and Technology, Karolinska Institutet, Stockholm, Sweden; 3 99018 Department of Infectious Diseases, Institute of Biomedicine, Gothenburg University, Gothenburg, Sweden; 4 Department of Clinical Microbiology, Sahlgrenska University Hospital, Region Västra Götaland, Gothenburg, Sweden

**Keywords:** Antisecretory Factor, breastfeeding, colostrum, human milk, lactation, milk bank, milk composition, neonatology, prematurity

## Abstract

**Background:**

Preterm infants are more susceptible to inflammatory complications than term infants. Human milk contains numerous bioactive components protecting the newborn infant. Antisecretory factor, a protein regulating secretory and inflammatory processes by complex binding with complement factors, is present in human milk.

**Research Aims:**

To describe antisecretory factor (1) in mother’s own milk in term and preterm infants; and (2) in donor milk before and after Holder pasteurization.

**Methods:**

The study was prospective, longitudinal, explorative, and descriptive. Antisecretory factor-compleasome was determined using sandwich enzyme-linked immunosorbent assay in longitudinal human milk samples over 12 weeks from mothers (*N* = 87) of term (*n* = 41) and of preterm (*n* = 46) infants and 20 anonymized donor human milk samples before and after Holder pasteurization.

**Results:**

Antisecretory factor-compleasome was overall higher in colostrum versus mature milk (*p* < .001) and no difference was found in term or preterm colostrum (*p* = .82). In mature milk, compleasome was higher and more variable in the preterm group (*p* = .01). After Holder pasteurization, compleasome levels increased (*p* < .001).

**Conclusion:**

Antisecretory factor followed the pattern of other immunological factors with high levels in colostrum. After preterm birth, levels of antisecretory factor were higher and more variable in mature milk. Holder pasteurization did not degrade antisecretory factor, indicating preserved anti-inflammatory properties in donor human milk.

## Abstract (sv)

**Bakgrund:**

För tidigt födda barn har en ökad risk att drabbas av inflammatoriska komplikationer i jämförelse med barn som är födda i fullgången tid. Bröstmjölk innehåller ett stort antal bioaktiva komponenter som skyddar det nyfödda barnet. Antisekretorisk faktor är ett protein som finns också i bröstmjölk och som reglerar sekretoriska och inflammatoriska processer genom att bilda komplex med komplementfaktorer,

**Syfte:**

Att genomföra en deskriptiv kohortstudie av antisekretorisk faktor i mammans egen bröstmjölk relaterat till prematur eller fullgången förlossning, samt studera antisekretorisk faktor i donerad bröstmjölk före och efter pastörisering.

**Metod:**

Studiens design var prospektiv, longitudinell och deskriptiv. Antisekretorisk faktor-kompleasom detekterades med enzymkopplad immunoadsoberande analys i longitudinella bröstmjölksprover insamlade under de första 12 veckorna efter partus från 87 mammor, 46 med prematurfödda barn och 41 med fullgångna barn, samt i 20 anonymiserade bröstmjölksprover från donator före och efter Holder pastörisering.

**Resultat:**

Antisekretorisk faktor-kompleasom var högre i kolostrum jämfört med i mogen bröstmjölk (*p* = .001) och i kolostrum var det ingen skillnad mellan prematur och fullgången grupp (*p* = .82). I mogen bröstmjölk var nivån av kompleasom högre och hade större variabilitet i prematurgruppen (*p* = .01). Efter Holder pastörisering ökade nivån av kompleasom i donerad bröstmjölk (*p* < .001).

**Konklusion:**

Antisekretorisk faktor följer samma mönster som många andra immunologiska faktorer med höga nivåer i kolostrum. Efter prematur förlossning var nivåerna av antisekretorisk faktor i mogen bröstmjölk högre och mer varierande. Holder pastörisering degraderade inte antisekretorisk faktor, vilket tyder på bevarade anti-inflammatoriska egenskaper i donerad bröstmjölk.

Back translated by Lisen Forsberg

## Background

Human milk contains a variety of biologically active components (Bardanzellu et al., 2020; Witkowska-Zimny & Kaminska-El-Hassan, 2017) and is involved in the development of the infant immune system and intestinal microbiota (Williams et al., 2019). Human milk has been suggested to reduce infant mortality, protect against infections, necrotizing enterocolitis, and other immunological diseases (Bardanzellu et al., 2020). Recently, researchers have detected a human milk microbiome (Moossavi et al., 2019), which suggests influence by factors like mode of delivery, duration of breastfeeding, as well as place of living. Human milk provides a multifactorial anti-inflammatory defense, including secretory IgA antibodies (Trend et al., 2016), directed particularly against the microbial flora of the mother. The composition of human milk varies over the lactation period, starting with colostrum, produced in low quantity and rich in immunologic components. Furthermore, in mothers who give birth preterm, the onset of lactation may be compromised, as well as the amount of milk and the composition (Moles et al., 2015; Trend et al., 2016), all of which may lead to a lack of availability of mother’s own milk (MOM) for the most vulnerable preterm infants. If MOM is not available, pasteurized donor human milk is often given. However, pasteurization affects many of the bioactive components in human milk and abolishes or reduces their activity (Rodríguez-Camejo et al., 2020).

Antisecretory factor (AF) is a 41 kDa endogenous protein regulating secretory processes and inflammation (Lange & Lönnroth, 2001). AF is present in most human tissues and body fluids, including human milk, probably mediating a protective role in the immune system. AF becomes activated as part of the innate immune response, for example following exposure to microbial pathogens (Johansson et al., 2018), forming a complex with complement factors, named compleasomes (Lönnroth et al., 2016). Endogenous plasma and human milk levels of active AF can be increased by enterotoxins and by certain food constituents (Johansson et al., 2019). Active AF can be induced in both plasma and human milk by oral intake of specially processed cereals (Lange & Lönnroth, 2001; Svensson et al., 2004) or by drinking spray-dried egg-yolk powder with a high content of AF-peptides (Zaman et al., 2018). Increase of endogenous AF synthesis improves the clinical outcome in diseases characterized by inflammation and secretory dysfunction (Zaman et al., 2018).

**Key messages**

- The immune regulatory protein, antisecretory factor, is described for the first time in longitudinal samples of human milk.
- Antisecretory factor is higher in colostrum than in mature human milk.
- Antisecretory factor in mature human milk is higher and more variable after preterm birth.
- Antisecretory factor is not destroyed after Holder pasteurization, suggesting preserved anti-inflammatory properties of donor milk.

Given the above, we concluded that human milk has important immunological and protective properties; infants born preterm are at risk for complications related to inflammation; and AF is an immune-regulating protein that has not, to our knowledge, previously been studied in human milk following term and preterm birth. As preterm infants often receive donor milk the influence of heating on the AF-compleasome is important to determine. Therefore, our aim was to describe antisecretory factor (1) in MOM in term and preterm infants; and (2) in donor milk before and after Holder pasteurization.

## Methods

### Research Design

This was a prospective, longitudinal, explorative, and descriptive study analyzing AF in human milk from a subgroup of mothers and infants included in the larger TELLUS-study (Telomer length, lung disease, and oxidative stress in preterm born infants), a cohort study of preterm infants with a healthy control group of term infants. The rationale for the study design was the lack of previous information on AF in the perinatal period and the possibility to collaborate in a larger cohort trial. The study was approved by the Regional Ethical Review Board in Stockholm, Sweden (Dnr 2014/921-32, Dnr 2018/1256-32).

### Setting and Relevant Context

The study was conducted in Stockholm, the capital of Sweden. The National Board of Health and Welfare Report (2019) stated that breastfeeding initiation rate in Sweden was 95%. At 6 months, 50% of mothers breastfed, 37% partially and 13% exclusively. Education and income was associated with a higher prevalence of breastfeeding during the first 6 months. According to the mothers’ country of birth, there were no differences in rates of exclusive breastfeeding; however, the rates of partial breastfeeding were higher in mothers born in non-European countries. Infants born term were breastfed in a higher proportion than preterm infants, both exclusively (at 6 months *OR* 0.58, CI [0.53, 0.63] and partially (at 6 months *OR* 0.8, CI [0.71,0.90]). In Stockholm, the breastfeeding rates were at initiation 96% and at 6 months 68%, with 56% partial and 12% exclusive breastfeeding.

Our hospital has approximately 4000–5000 deliveries per year. Rooming in is routine care at the postnatal ward and the average hospital stay is 2 days after vaginal delivery and 3 days after caesarean section. The neonatal intensive care unit (NICU) has 18 beds and uses a family centered approach, allowing rooming in for parents. For preterm infants in the NICU, pasteurized donor human milk is given up to 35 weeks of gestation when MOM is not available or the volume is not reaching the infant’s needs.

### Sample

Our target population was mothers of preterm infants, born < 30^0^ weeks^days^ of gestation who were available for inclusion. Mothers of term infants (37^0^ – 41^6^ weeks^days^ of gestation) were recruited as controls. Exclusion criteria were mothers with infants born with severe malformation. A total of 181 infant–mother pairs consented for participation in the TELLUS study during the time period. The total sample size was 87 participants, 41 in the term group and 46 in the preterm group. In the preterm group, one participant had triplets and 14 participants had twins (for one participant of twins only one infant was live born), which is why the total number of infants was 102 in the study, 41 in the term group, and 61 in the preterm group. We estimated a sample of approximately 40 mothers in each group to be sufficient for this exploratory study.

Human milk donors are often mothers of term infants who express milk and donate to the milk bank, but human milk can also be donated by mothers of preterm infants with excessive milk production. Lactating women can donate human milk for 3 months postpartum, after submitting a health declaration and a blood test for some infectious diseases. In Stockholm donor human milk is not pooled, that is, mixed from multiple donors. The human milk from donors is kept separate with a single donor in each batch. We estimated a sample of human milk from 20 different mothers to be sufficient as a pilot sample for this explorative analysis to demonstrate an effect of Holder pasteurization.

### Measurement

A sandwich enzyme-linked immunosorbent assay (ELISA) for the detection of compleasomes (proteasome/complement complexes) in the pre-treated human milk was performed as previously described for plasma (Lönnroth et al., 2016). The biological activity of AF-compleasome had been tested during the development of the in vitro method using AF-monoclonal antibodies. The samples were also tested in vivo (rat loop model) and the two methods were shown to correlate (Johansson et al., 2009). In house produced monoclonal antibodies (mAbs) against proteasome subunit AF (Johansson et al., 2009) diluted 1:200 or phosphate-buffered saline (PBS) as control, were coated on 96-well Maxisorp microtiter plates overnight (Fisher Scientific, GTF AB, Gothenburg, Sweden). After blocking with 0.2% bovine serum albumin (BSA) at 37 °C for 45 min, human milk samples were titrated in PBS with 0.2% BSA and 0.05% Tween 20 and shaken for 2 hr at RT. Polyclonal antibodies against C3c at 1:2000 dilution was used as the detecting antibody (Dako, Glostrup, Denmark). An anti-rabbit-alkaline phosphatase (AP)-conjugated antibody was applied after 1 hr of incubation (Jackson ImmunoReasearch Europe Ltd., Västra Frölunda, Sweden) and AP substrate was added after a further hour. Absorbance was read at 405 nm in a photometer (Emax precision microplate reader, Molecular Devices, Sunnyvale CA, US), and the difference between antibody-coated samples and controls (PBS) was estimated. For intra-assay quality control, samples were analyzed in duplicate and a positive control with known high AF levels was used. The measurement units of AF-compleasome were net-Abs 405 nm.

### Data Collection

Anonymized donor human milk was collected at the milk bank during March 2019. Samples of mothers’ own milk were collected longitudinally at Weeks 1, 4 (preterms only), and 12, between April 2014 and April 2019. All mothers received written and oral information about the study and signed informed consent forms. Samples of MOM were collected by members in the TELLUS research group in the NICU or postnatal ward during the hospital stay, or at check-up visits after discharge. Each participant was given an individual study ID, used on all samples, and collected data in the database to maintain confidentiality.

The collected samples of MOM were predominantly expressed during daytime, in near proximity to collection of other samples in the study. The milk could be hand expressed (most mothers of term infants) or expressed with a breast pump (most mothers of preterm infants). For term mothers at sampling time point Weeks 1 and 12 postpartum, the timing of expression could be done before or after a breastfeed, and that could also be the case for mothers of preterm infants at 12 weeks. After collection, the samples of human milk were centrifuged at 2000 g for 15 min at room temperature (RT) and the fat- and cell-free middle phase was collected in cryo vials and kept at -80 °C until analysis was performed. Human milk samples (5 ml) from 20 anonymized donors, were divided into two tubes. One was directly stored in a -20 °C freezer, and the other was pasteurized by Holder pasteurization performed at 62.5 °C for 30 min (Moro et al., 2019) and then stored in a -20 °C freezer. After collection of all samples within a week, the samples were transported to a -80 °C freezer for storage until analysis. The human milk was pre-treated with ethyl acetate (> 99.5% pure) before the ELISA analysis. Milk samples were mixed with equal portions of ethyl acetate, for removal of disturbing fat in the ELISA analysis, and shaken in 5 min, after which the milk-ethyl acetate mixture was centrifuged at 1000 g for 5 min at RT. The fat-free sub-phase was collected for further analysis.

### Data Analysis

AF-compleasome in MOM was analyzed in relation to demographics using independent *t*-test and Pearson correlation (Aim 1). A *p*-value < .05 was regarded as statistically significant. We analyzed AF-compleasome in MOM in relation to term and preterm birth, time point of sampling and longitudinally, using Pearson correlation, independent *t*-test, two-samples *t*-test with unequal variances, and paired *t*-test. The *p*-values were calculated using a two-sample *t*-test with unequal variances for the differences between the groups, and Generalized Least Squares (GLS) random effects model to test the within-group differences between timepoints. A random effects model was used to take into consideration that there were repeated measures, but that not everyone was measured at all timepoints. To compare the distributions between term and preterm in longitudinal samples, we used kernel densities, a non-parametric method to approximate distributions based on data.

The difference between AF-compleasome in donor human milk before and after Holder pasteurization (Aim 2) was analyzed using independent *t*-test and paired *t*-test. For analysis and figures, the software SPSS (Version 26) and Stata (Version 15) were used.

## Results

### Characteristics of the Sample

Characteristics of mothers and infants at entry of the study are presented in Table 1. Maternal age and body mass index (BMI) were similar in the term and preterm group. The mean gestational age for term infants was 39^6^ weeks^days^ and for preterm infants it was 27^2^. In term infants the birth weight was *M* = 3614 (*SD* = 448) grams; in preterm infants it was *M* = 1014 (*SD* = 241). Among the term infants 21 (51%) were females, while among the preterm infants (*n* = 61) 34 (56%) were female.

**Table 1:** Characteristics of Participants Grouped by Infants’ Gestational Age Category (*N* = 87).

Characteristic	Term *n* = 41(47.1%) n (%)	Preterm *n* = 46 (52.9%) n (%)
Primiparous	22 (54)	30 (65)
Multiple pregnancy	0 (0)	15 (33)
Vaginal delivery	31 (76)	14 (30)
Cesarean section	10 (24)	32 (70)

*Note*. In the preterm group, one participant had triplets and 14 participants had twins,for one participant of twins only one infant was live born

### AF-Compleasome Levels (Aim 1)

Human milk was collected during the 1st week (colostrum), at 4 weeks (mothers of preterm only) and at 12 weeks (mature milk) postpartum. No more than one sample was collected at each timepoint. All included participants were not able to express their milk at all timepoints; some only expressed milk at one timepoint. A total of 128 samples of human milk were analyzed for the content of AF-compleasome, of which 48 samples were from participants with term infants and 80 samples were from participants with preterm infants. The number of samples at each timepoint (and the missing values) are presented in Table 2 and postnatal day for sampling at each timepoint is presented in Table 3.

**Table 2:** Number of Milk Samples Collected at Each Timepoint Grouped by Gestational Age Category (*N* = 87).

Timepoint	Term *n* = 41 (47.1%) *n (%*)	Preterm *n* = 46 (52.9%) *n (%*)
Week 1	22 (53)	40 (87)
Week 4	0 (0)	15 (33)
Week 12	26 (63)	25 (54)
Longitudinal samples	7 (17)	17 (37)

*Note*. Missing values: Week 1 = 19 term and 6 preterm; Week 4 = 31; Week 12 = 15 term and 21 preterm; longitudinal samples = 34 term and 29 preterm.

**Table 3:** The Mean (*SD*) of Collected Milk Samples Grouped by Gestational Age Category (*N* = 87).

	Term *n* = 41(47.1%)	Preterm *n* = 46 (52.9%)
Time after birth	*M (SD*)	*M (SD*)
Week 1	3 (2)	7 (4)
Week 4		33 (8)
Week 12	104 (38)	99 (37)

*Note*. A significant difference (*p* = < .01) was found between groups during Week 1. Missing values: Week 1= 19 term and 6 preterm; Week 4 = 4 term and 31 preterm; Week 12 = 15 term and 21 preterm.

**Figure 1:**
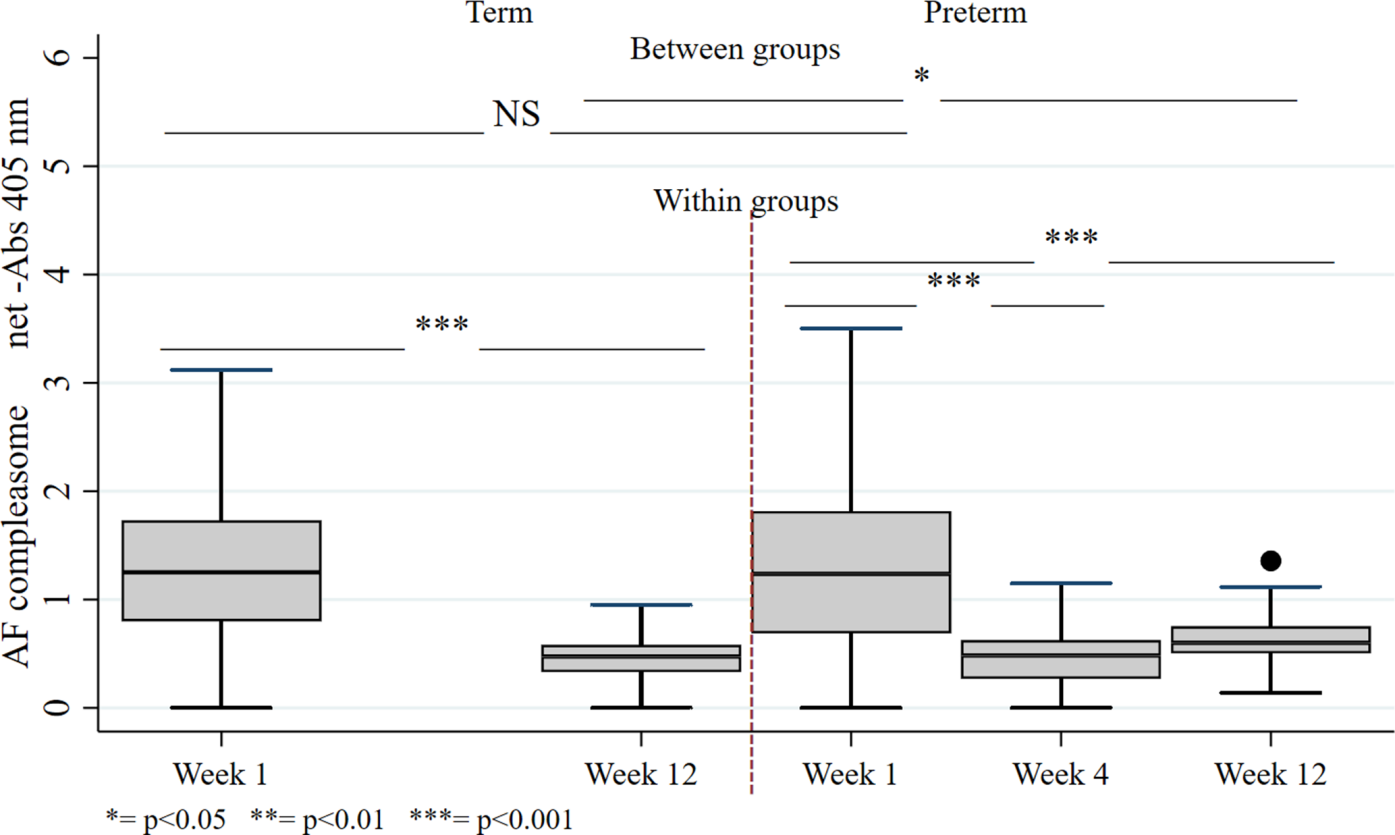
AF-compleasome in Mothers’ Own Milk After Term or Preterm Birth.

AF-compleasome was present in all samples, and the measurement units of AF-compleasome were net-Abs 405 nm. Overall, in samples from both the term and preterm group, AF-compleasome levels in human milk were higher in colostrum than in mature milk (*M* = 1.32, *SD* = 0.68 vs. *M* = 0.54, *SD* = 0.21; *p* < .001). The AF-compleasome levels were similar in colostrum (Week 1) for both groups (*M* = 1.34, *SD* = 0.71 vs. *M* = 1.30, *SD* = 0.64, *p* = .82), but higher in mature human milk (Week 12) from mothers of preterm infants (*M* = 0.62, *SD* = 0.24 vs. M = 0.47, *SD* = 0.15; *p* < ..05), and changes over time shown in Figure 1. Postnatal day for sample collection varied between samples (Table 3) and at Week 1 there was a significant difference between groups. There was a negative correlation between AF-compleasome and postnatal day for sample collection (*r* = −.496, *p* < .001). However, when excluding samples collected after the first seven postnatal days (preterm group, *n* = 11, range day 8–16), there was no correlation between AF-compleasome and day of sample collection (*r* = −.155, *p* = .42). There was no correlation between AF-compleasome and postnatal day for sample collection at 12 weeks (*r* = −.156, *p* =.58).

#### Levels in Longitudinal Samples

**Figure 2:**
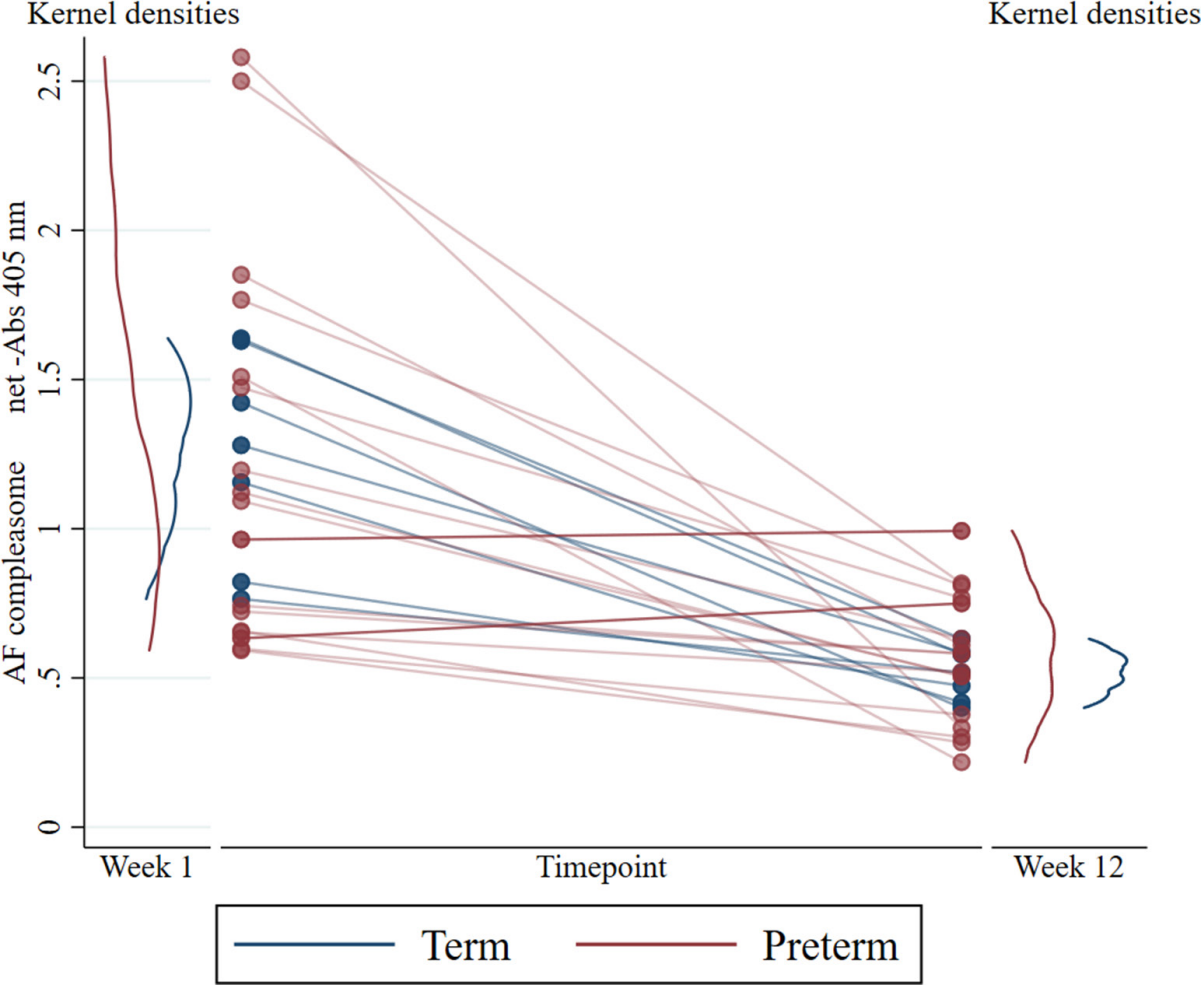
AF-Compleasome Changes and Variations Over Time in Mothers’ Own Milk. Detection of AF-Compleasome in Longitudinal Human Milk Samples Taken From Mothers After Term or Preterm Birth Using Sandwich Enzyme-Linked Immunosorbent Assay (ELISA).

We also investigated the formation of AF-compleasome over time in milk from individual mothers where samples were obtained at all timepoints. Longitudinal human milk samples were collected from 17 (37%) mothers of preterm infants and 7 (17%) mothers of term infants (Week 1 and Week 12). Higher AF-compleasome levels were demonstrated at Week 1 versus Week 12 in both the preterm group (*M* =1.21, *SD* = 0.64 vs. *M* = 0.56, *SD* = 0.22, *p* < .01) and term group (*M* =1.24, *SD* = 0.35 vs. *M* = 0.51, *SD* = 0.09, *p* < .01). However, human milk samples from two mothers in the preterm group exhibited similar or slightly higher AF-compleasome at the later timepoint, lines marked with darker color in Figure 2 (Week 1 vs. Week 12; 0.63 vs. 0.75 and 0.96 vs. 0.99 respectively). Kernel densities demonstrate a similar distribution between groups at Week 1, but a wider distribution in preterm versus term at Week 12 (Figure 2). In longitudinal samples of human milk from preterm mothers with samples from Weeks 1, 4 and 12 (*n* = 15), AF-compleasome levels were high Week 1, had decreased already at 4 weeks (*M* = 1.24, *SD* = 0.67 vs. *M* = 0.44, *SD* = 0.21, *p* < .001) *p* < .001) and remained low at postnatal Week 12 (*M* = 1.24, *SD* = 0.67 vs. *M* = 0.54, *SD* = 0.22; *p* < .001 vs. Week 1).

#### AF-Compleasome Levels Related to Maternal and Infant Characteristics

For the total group, as well as the term and preterm groups respectively, there were no correlations at any timepoint between AF-compleasome levels in the milk and maternal age or body mass index. Participants with term infants, who were multiparas had higher AF-compleasome in their milk in Week 1 versus participants who were primiparas (*M* = 1.58, *SD* = .58 vs. *M* = 1.01, *SD* .58 *p* = .03) but there was no difference within the preterm group on this variable at any measurement point. There were no differences in AF-compleasome based on mode of delivery or infant sex in either group or as a total group.

### AF-Compleasome Levels in Donor Milk, Before and After Pasteurization (Aim 2)

**Figure 3:**
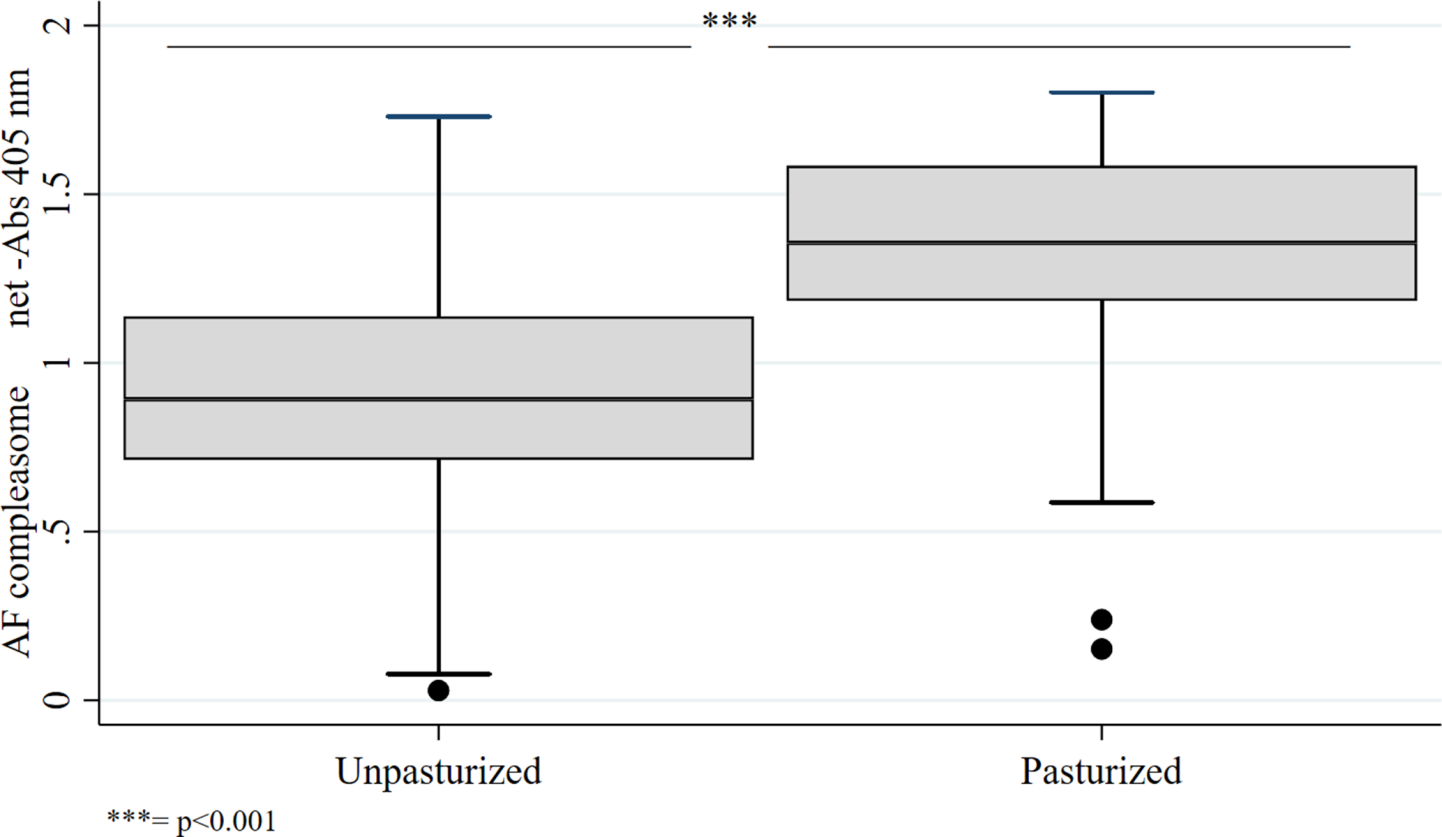
Detection of AF-compleasome in donor human milk, before and after Holder pasteurization using sandwich enzyme-linked immunosorbent assay (ELISA).

Anonymized donor milk samples (*n* = 20), were collected at the milk bank at Södersjukhuset, Stockholm. Donor human milk demonstrated higher AF-compleasome levels after Holder pasteurization than before pasteurization (*M* = 1.30, *SD* = 0.44 vs. *M* = 0.91, *SD* = 0.45 *p* < .001; Figure 3).

## Discussion

Here we present the results from the very first in vitro analysis of human milk using the combination of antibodies against AF together with detecting antibodies against complement Factor 3. AF-compleasome was present in all human milk samples and demonstrated an inter-individual variation of level. Preterm birth did not change the pattern of AF-compleasome levels in human milk declining over the first weeks postpartum. Interestingly, there was no difference in AF-compleasome in colostrum related to term and preterm birth, but the levels were higher and more variable in mature human milk from preterm mothers. AF-compleasome decreased already at 4 weeks in relation to the 1st week postpartum. These results are in line with a higher level of most immunological factors in colostrum than in mature milk with a role to protect the newborn infant exposed to a great number of environmental factors after birth (Bardanzellu et al., 2020; Moles et al., 2015; Sahin et al., 2020). Furthermore, heat treatment of donor human milk by Holder pasteurization increased AF-compleasome after pasteurization, suggesting a sustained anti-inflammatory effect even though many bioactive components are known to be reduced or destroyed (Rodríguez-Camejo et al., 2020).

The composition of human milk changes during lactation—both macronutrients and bioactive factors—with higher levels of many components in preterm milk (Moles et al., 2015; Trend et al., 2016). However, a recent study demonstrated higher fat and carbohydrate levels on Day 28 after birth, and higher energy levels on Days 14 and 28, but no difference in protein content in human milk from mothers of term infants versus mothers of preterm infants (Sahin et al., 2020). Their results demonstrated significant changes in macronutrient content over time during lactation for both the preterm and term group. In the present study, we demonstrate higher levels of AF-compleasome in colostrum than in mature human milk, which is in line with many other immunological components in human milk (Trend et al., 2016), but no difference in AF-compleasome levels in colostrum after term and preterm birth. However, longitudinal samples of milk from two mothers of preterm infants displayed similar or slightly higher AF levels over time. The wider variability and different pattern may be due to the greater heterogeneity of mothers in the preterm group related to the higher prevalence of pregnancy complications as preeclampsia, infections, delivery by acute caesarean section, as well as other factors like diet shown to affect milk composition in other studies (Moossavi et al., 2019). The result with high levels of AF in colostrum after both term and preterm birth differs from our previous findings of lower AF levels in placenta after preterm birth (Gustafsson et al., 2018) and may suggest a programming of high levels in colostrum due to the exposure to inflammatory factors for both term and preterm infants immediately after birth. Preterm birth is associated with proteins linked to inflammation (Olin et al., 2018; Zhong et al., 2021). Our results are in line with the findings of a more variable metabolic profile in human milk after preterm birth during the first 4 weeks postpartum (Spevacek et al., 2015). A previous study demonstrated lower levels of host-defense proteins, as complement C3, in human milk from mothers of very preterm infants versus milk from mothers of later preterm and term infants (Broadhurst et al., 2015). Their results also demonstrated decreasing levels from postnatal Weeks 2–5, except for the very preterm group of which the host defense protein levels remained elevated at the later time-point of lactation. The mechanism regulating the AF-compleasome during lactation may be related both to the mother and to the infant, as demonstrated for other bioactive components in human milk (Hassiotou & Geddes, 2015), and needs further investigation.

Several researchers have demonstrated a greater variability in the levels of most immunological factors in human milk from mothers of preterm versus term infants, suggesting there may be a compensatory mechanism with immunological adaption following preterm birth (Bardanzellu et al., 2020; Spevacek et al., 2015). However, Broadhurst et al. (2015) found lower levels of host-defense proteins in human milk of mothers of very preterm infants, indicating that the lactogenic compensatory mechanisms for development of the immature human milk may not be fully effective following very short gestational length. We need to better understand the biological mechanisms of whether high AF-compleasome levels are due to exposure to inflammation and an action to regulate and control tissue damage, or if higher AF-compleasome levels are associated with a biological preventive effect on inflammation.

A small proof-of-concept study of the human milk proteome has described higher abundance of secretory IgA and IgM in transitional milk compared to higher IgG in mature milk, indicating a change in milk function over time, with focus on direct pathogen killing in the early lactation, shifting towards antigen intake that aims to develop the infant’s immune system later in lactation (Zhu et al., 2021). Since AF binds to C3, it may also be a part of the regulation of the complement system, with a down-regulating influence on inflammatory processes (Lönnroth et al., 2016).

The analysis of the anonymized human milk from donors demonstrated higher AF levels after Holder pasteurization compared to the analysis done before. This was an unexpected result since pasteurization is known to abolish or reduce many of the bioactive components in human milk (Rodríguez-Camejo et al., 2020). AF is activated in complex with complement factor C3, converting C3 to the inactive form C3c (Lönnroth et al., 2016). Heating human milk to 56 ℃ has been described to inhibit complement activation (Maye et al., 2015). Therefore, the heating during Holder pasteurization could potentially contribute to formation of more activated AF-compleasome. Furthermore, the ability of human milk to inhibit complement activation has been proposed as a mechanism behind the protective properties to reduce the risk of inflammatory-induced tissue damage (Zhu et al., 2021). Higher AF levels in donor human milk after pasteurization may hypothetically help to protect the infant from inflammation, and consequently be a part of an explanation for why pasteurized human milk still seems to have a positive effect on reducing the risk for necrotizing enterocolitis (Quigley et al., 2019) for preterm infants when MOM is not available, even though many of the other immunological bioactive and bactericidal components are destroyed or inactivated (Rodríguez-Camejo et al., 2020). In animals, the biological effect of induced AF levels in milk has been described and shown to result in less diarrheal diseases, increased survival, and higher weight gain in the offspring (Lange & Lönnroth, 2001).

Our findings warrant further study of AF in human milk and the possible influence on maternal clinical and/or subclinical mastitis, milk supply and composition, as well as the potential effect for preterm infants related to inflammatory complications, growth, and health.

### Limitations

In the present study, there was a difference between groups for sample collection at the first timepoint, Week 1. However, since the onset of secretory activation is often delayed after preterm birth (Parker et al., 2019), this may be reflected in the later collection of samples in the preterm group. Longitudinal analysis demonstrated lower AF-complesome at 4 weeks versus at 1 and 12 weeks postpartum, but we did not have samples from mothers of term infants at 4 weeks to compare whether AF-compleasome levels decreased with a similar pattern. Unfortunately, we did not have longitudinal samples from all participants due to difficulties expressing milk at the earlier timepoint or not still providing milk for the infant at the later timepoint. The resulting large number of missing values for specific time points is an unavoidable limitation in our clinical study, however, we believe that the explorative design and the novelty of AF-compleasome in human milk makes the description valid. The presence of multiples in the preterm infant group resulted in a small number imbalance, but reflect the clinical nature of our study as preterm birth is more common in multiple pregnancies. Unfortunately, we did not have information about maternal diet which may have influenced individual mothers’ AF levels, and further studies are needed to describe the possible effect of different diets.

### Conclusion

Following birth, levels of active AF-compleasome appear to be higher in colostrum than in mature milk. The difference is in line with higher levels of other immunological factors in human milk. High levels of AF-compleasome in colostrum may have a role in protection against inflammatory processes after birth. A more variable level of AF-compleasome in the mature human milk of preterm mothers may suggest a response to greater inflammatory stimuli in the environment. In donor human milk, we found higher AF-compleasome levels after pasteurization than before, suggesting preserved anti-inflammatory effect also in donor milk. This is an important finding supporting the use of donor human milk in neonatal intensive care units as an alternative when MOM is not available.
